# Demineralized Bone Matrix Carriers and their Clinical Applications: An Overview

**DOI:** 10.1111/os.12509

**Published:** 2019-09-08

**Authors:** Hao Zhang, Li Yang, Xiong‐gang Yang, Feng Wang, Jiang‐tao Feng, Kun‐chi Hua, Qi Li, Yong‐cheng Hu

**Affiliations:** ^1^ Department of Bone Tumor Tianjin Hospital Tianjin China; ^2^ Graduate School Tianjin Medical University Tianjin China; ^3^ Beijing Wonderful Medical Biomaterial Co. Ltd. Beijing China

**Keywords:** Bone graft, Bone regeneration, Demineralized bone matrix, Viscous carriers

## Abstract

Reconstruction of massive bone defects is challenging for orthopaedic clinicians, especially in cases of severe trauma and resection of tumors in various locales. Autologous iliac crest bone graft (ICBG) is the “gold standard” for bone grafting. However, the limited availability and complications at donor sites resulted in seeking other options like allografts and bone graft substitutes. Demineralized bone matrix (DBM) is a form of allograft using acidic solution to remove mineral components, while leaving much of the proteinaceous components native to bone, with small amounts of calcium‐based solids, inorganic phosphates, and some trace cell debris. It is an osteoconductive and osteoinductive biomaterial and is approved as a medical device for use in bone defects and spinal fusion. To pack consistently into the defect sites and stay firmly in the filling parts, DBM products have various forms combined with biocompatible viscous carriers, including sponges, strips, injectable putty, paste, and paste infused with chips. The present review aims to summarize the properties of various kind of viscous carriers and their clinical use combined with DBM in commercially available products. Given DBM'mercially available products. Given DBM;s long clinical track record and commercial accessibility in standard forms, opportunities to further develop and validate DBM as a versatile bone biomaterial in orthopaedic repair and regenerative medicine contexts are attractive.

## Introduction

Reconstruction of massive bone defects is challenging for orthopaedic clinicians, especially in cases of severe trauma and resection of tumors in various locales. Many bone graft materials have been applied to accomplish this procedure, including autogenous bone, allogeneic bone, xenogenic bone, and other non‐bone derived substances. In general, an ideal bone substitute material should be equipped with the following three elements: (i) “osteoconductivity,” acting as the “soil,” the three‐dimensional process of ingrowth of sprouting capillaries, perivascular tissue, and osteoprogenitor cells from the recipient bed into the structure of an implant or bone graft^1^; (ii) “osteoinductivity,” acting as the “fertilizer,” the process of differentiation of pluripotential mesenchymal cells into osteoprogenitor cells and ultimately into osteoblasts that form bone as a consequence of a stimulating agent (i.e. bone morphogenetic protein)^2^; (iii) “osteogenesis,” acting as the “seed,” the ability to reconstruct tissue to form new bone tissue, which refers to the presence of osteoprogenitor cells that directly promote new bone growth at the transplant site. Among the multiple reconstruction substitutes, bone autograft, mainly referring to the iliac crest bone graft (ICBG), has been universally recognized as the “gold standard,” because it possesses the aforementioned elements simultaneously^3^. Despite this, a notable concern about autografts is the invasive “donor” procedure required to harvest the graft, which tends to result in more postoperative complications, such as severe pain and infection at the donor site^4^. Moreover, elevated time on the operating room table, increased patient care cost, inadequate donor bone, and mismatched bone shape with the recipient site also reveal that autografts are not an absolutely perfect transplant material^5^. Therefore, clinicians have been devoted to exploring alternatives to autografting, to overcome the drawbacks of autografts.

Demineralized bone matrix (DBM) has been developed as an allogeneic alternative to autografting, which is an important therapeutic option for appendicular, axial, and craniofacial bone defects. It is a decalcified product using acidic solution to remove mineral components, while leaving behind collagen (mainly type I with some types IV and X), non‐collagen proteins, some osteoinductive growth factors (e.g. bone morphogenic proteins, BMP), variable percentages of residual calcium phosphate mineral (1%–6%), and some small percentages of cellular debris^6^. After decalcification, BMP could be released from the surrounding mineral components and fully exert its osteoinductive potential^7^. The remaining collagen proteins in DBM could provide a 3D configuration for ingrowth of host capillaries, perivascular tissue, and osteoprogenitor cells into the graft. Thus, the DBM has been demonstrated to be an osteoconductive and osteoinductive substitute. In the meantime, the original cells and possible bacteria in the allogeneic bone are killed, which could reduce the risks of immune rejection and infection. DBM used for bone reconstruction has many advantages: (i) it is not limited by graft amount, as the donor source is abundant; (ii) it could reduce complications of autograft harvest at the donor site; and (iii) it could shorten the operation and recovery time.

**Figure 1:**
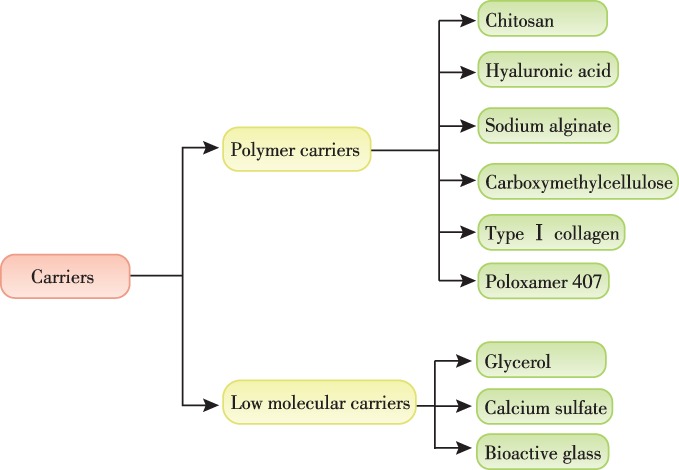
The common viscous carriers could be divided into two classifications according to molecular weight.

The use of DBM can be traced back to 1889 when Sen^8^. first used DBM derived from oxen tibiae to repair the skull and long bone defects in humans. Then, the human‐derived DBM was successfully transplanted for human long bone defects and lumbar spine by Urist *et al*.^9^ in 1965, for the first time. Therefrom, DBM has been used more frequently in orthopaedic surgeries, and plenty of research has been carried out to explore the bone regeneration capacity of DBM as a bone substitute material. Although DBM has been widely proved to have attractive osteoinductive and osteoconductive potential, but the end products, of various forms, are not easy for clinicians to manage: (i) the powder or particles of DBM can be loose in structure and may not stay firmly in the filling site, and could be easily dispersed by irrigation and blood flow during surgery; and (ii) the application of pre‐formed materials for filling may leave a dead space due to the inconsistency of the repair material and the shape of the defect, resulting in non‐union or delayed healing^6^. Hence, currently the most popular DBM product is a moldable bone paste or putty, which can be consistently packed into defect sites and remain firm. A moldable DBM product could be made of the composite of DBM powder/particles combined with biocompatible viscous carriers, which provides a stable suspension of DBM powder/particles. In general, according to their molecular weight, the viscous carriers could be divided into the following two classifications: (i) polymer materials, such as collagen, chitosan, hyaluronic acid (HA), carboxymethylcellulose (CMC), and poloxamer 407; and (ii) low molecular materials, such as glycerol, calcium sulfate, and bioactive glass (Fig. 1).

This review aims to summarize the properties of various viscous carriers and their applications combined with DBM in commercially available products.

## Study Searching and Selecting

To identify studies concerning DBM carriers and their clinical applications, a systematic literature search was performed. We searched electronic platforms including PubMed, Embase, and Cochrane Library from inception to 25 March 2018, and the language was restricted to English. Search terms included “carrier name” and “demineralized bone matrix.” A combined search using the subject terms (Medical Subject Heading, MeSH) and free terms was carried out in every database. Reference lists of the included studies were also viewed for any additional papers. In addition, we consulted related companies for additional published or unpublished studies.

Two authors independently selected studies following the predetermined selection criteria; any disagreement was resolved by discussion. EndNote X8 (Clarivate Analytics, Philadelphia, USA) was used to detect and merge the duplicates, and then titles and abstracts were evaluated to identify the ones that met the criteria. Finally, full texts were reviewed for inclusion.

**Figure 2:**
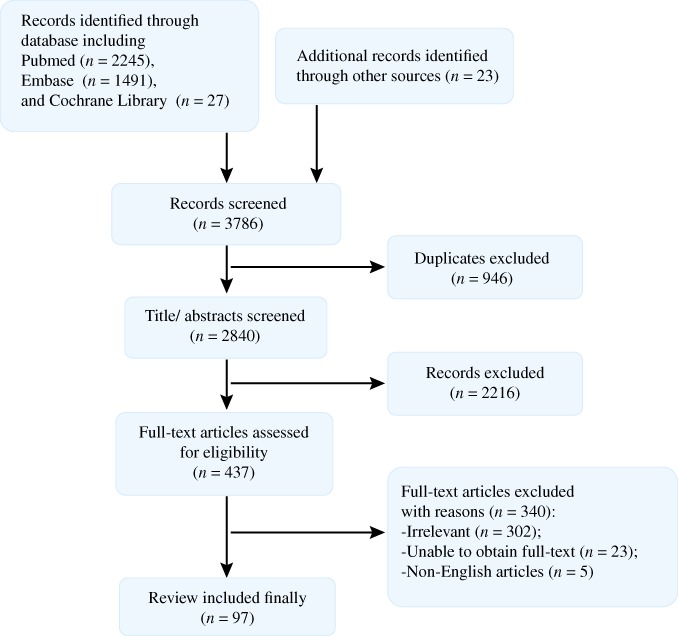
Literature searching process. A total of 3786 articles are identified from databases, including PubMed, Embase, and Cochrane Library. After removing duplicates and screening all titles and abstracts, 437 articles were excluded. Then full texts were read carefully, and, finally, 97 articles were included.

A total of 3458 potentially relevant articles were identified. After removing duplicates (1476 articles) by using EndNote X8 and screening all titles and abstracts, 897 articles were excluded. Then full texts were read carefully, and, finally, 97 articles were included. The literature search process is presented in Fig. 2.

## Demineralized Bone Matrix Carriers

Generally, a viscous carrier should be equipped with the following characteristics: (i) *Good biocompatibility with the tissues*: nontoxicity, nonimmunogenicity, nonteratogenicity, and noncarcinogenicity; (ii) *Proper biodegradation rate*: corresponding to the osteogenesis procedure, to ensure the successful repairing of the bony defect; (iii) *Viscidity and plasticity*: assuring graft could be shaped as necessary and stay firmly in filling site; (iv) *Osteoconductive three‐dimensional microstructure*: promoting ingrowth of host capillaries, perivascular tissue, and osteoprogenitor cells into the graft; and (v) O*ptimal mechanical property* 10. Satisfying the above conditions, a variety of carrier materials have been selected to compound the DBM to deliver a plastic decalcified bone paste, and these carriers include the polymer materials and the low molecular materials according to their molecular weight.

### *Polymer Carriers Materials*

The polymer carriers mainly include the natural polymer materials (e.g. chitosan, HA, sodium alginate, CMC, and type I collagen) and synthetic polymer materials (e.g. poloxamer 407). They are all demonstrated to be biocompatible and biodegradable after implantation *in vivo*, and have attractive viscidity to cohere with DBM powder particles.

#### *Chitosan*

**Figure 3:**
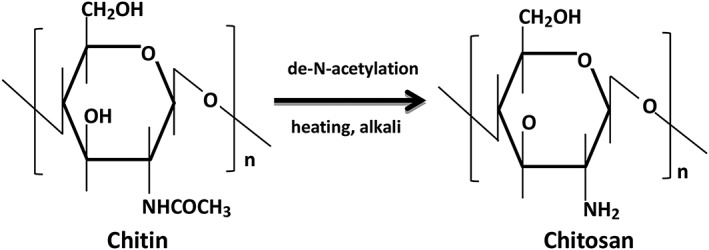
Chemical structure of chitin and chitosan, and the de‐N‐acetylation process from chitin to chitosan.

The history of chitosan could be retrospected to 1859 when Rouget first heated chitin to the boiling point in a concentrated KOH solution^11^. Chitin, the source material for chitosan, is the major component of the exoskeleton of invertebrates, crustaceans and insects, and the cell wall of fungi and yeast, in which it acts as a supportive and protective structure^12^. Chitosan is a linear polysaccharide and is composed of glucosamine and N‐acetyl‐glucosamine units linked by β (1‐4) glycosidic bonds^10^. In general, when the percent of N‐acetyl‐glucosamine units is higher than 50%, the biopolymer should be termed chitin, and vice versa. As represented in Fig. 3, the chitosan is a product obtained from the de‐N‐acetylation of chitin, in the presence of hot alkali. The content of glucosamine in chitosan molecules is defined as the degree of deacetylation (DD). The DD and source of chitin may obviously influence the molecular weight of chitosan, which ranges from 300 to over 1000 kDa, with a DD of 30%–95%^13^.

Chitosan could not be dissolved in neutral or alkaline solution but could be easily dissolved in dilute acid solution (pH < 6, such as dilute acetic acid), where the free amino groups are protonated and the molecule becomes soluble^14^. Porous chitosan structures can be formed by freezing and lyophilizing chitosan acetic acid solutions in suitable molds. Porous materials play a significant role in the bone implantation process.

It has been widely recognized that the chitosan is biocompatible and biodegradable *in vivo*, and its degradation products are non‐toxic, non‐immunogenic, and non‐carcinogenic^15^. Lysozyme is the primary enzyme that is responsible for degradation of chitosan *in vivo*, which appears to target acetylated residues^16^. The final degradation products are biocompatible chitosan oligosaccharides with variable length. The biodegradation rate of chitosan may be diverse among biopolymers with different DD, as it would influence the degree of crystallinity. It has been reported that highly deacetylated forms of chitosan (>85%) exhibit a relatively slow degradation rate that may last several months, while the forms with lower DD degrade more rapidly^17^.

Chitosan is a unique natural animal cellulin with positively charged cation. The cationic nature of chitosan is responsible for attracting various negatively charged proteoglycans, which helps the osteoblasts’ adhesion to it. In addition, Chitosan has antibacterial activity^18^, and antifungal^19^, mucoadhesive^20^, analgesic^19^, and hemostatic properties^21^, and the ability to promote osteogenic progenitor cell recruitment and attachment, thus facilitating bone formation^22^. It can be produced in various forms, including hydrogel, powder, small sphere, tablet, capsule, microbead, particulate, sponge, nano‐fiber, and textile fiber. Therefore, this unique biopolymer is an outstanding candidate for biomedical applications, especially for tissue engineering.

#### *Hyaluronic Acid*

**Figure 4:**
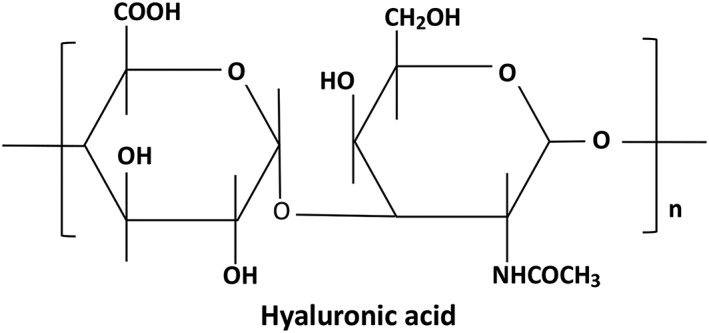
Chemical structure of hyaluronic acid, which is composed of N‐acetyl‐D glucosamine and D‐glucuronic acid. D‐glucuronic acid and N‐acetylglucosamine are linked by a β‐1,3‐glycosidic bond, and the disaccharide units are linked by a β‐1,4‐glycosidic bond.

Hyaluronic acid (HA) is a unique linear macromolecule acidic mucopolysaccharide composed of N‐acetyl‐D glucosamine and D‐glucuronic acid. D‐glucuronic acid and N‐acetylglucosamine are linked by a β‐1,3‐glycosidic bond, and the disaccharide units are linked by a β‐1,4‐glycosidic bond. The two monosaccharides in the molecule are composed in a molar ratio of 1:1. The chemical structural of HA is shown in Fig. 4. HA molecules contain a large amount of carboxyl groups and hydroxyl groups, forming intramolecular and intermolecular hydrogen bonds in aqueous solution, so they have strong water retention, and can bind more than 400 times of water at higher concentrations, because the complex three‐stage network structure formed by its intermolecular action has an aqueous solution with remarkable viscoelasticity.

The polysaccharide chains of HA are linear and unbranched and roll up into a coil conformation. The irregular crimping state and hydrodynamics of HA in solution give it many important physical properties, such as high degree of viscoelasticity, moldability, degradability, permeability, and good biocompatibility^23^.

The presence of HA in an aqueous solution shows a complex rheological behavior, including this system into the pseudoplastic fluids，which is very important for its applications. The rheological characterization of HA aqueous solutions has been carried out byGarcia^24^, determining the value of the intrinsic viscosity and the average molecular weight. Both the increase of temperature and the presence of an electrolyte produce an important decrease in the viscosity magnitude, as well as an approximation to the Newtonian behavior related to its rheology. Procedures for introducing covalent cross‐links in hyaluronan matrices have been developed to create stable networks and semisolid materials exhibiting pronounced viscoelastic properties^25^.

Hyaluronic acid widely exists in human tissues such as joints, vitreous bodies, synovial fluid, cartilage, skin, and other tissues and organs as a major constituent of the extracellular matrix (ECM). It has been reported to play an important role in tissue repair and regeneration^26^. Moreover, it has been demonstrated that HA hydrogels could retain BMP‐2^27^. HA has also been found to affect the interplay between osteoclasts and osteoblasts that is important in bone remodeling and fracture healing^28^.

Most of the HA absorbed by the human body enters the lymph nodes and degrades into monosaccharides in the lymph nodes, and a small part enters the blood circulation. Approximately 80% of HA in the blood circulation is quickly ingested by the liver, and the rest is handled by the spleen and other organs. The final product of HA metabolism in the body is carbon dioxide and water, which has no toxic side effects on the body.

#### *Sodium Alginate*

Alginate is the most abundant marine biopolymer, which comprises a rather broad family of polysaccharides found in brown seaweeds (Laminariasp., Macrocystis sp., Lessonia sp., and others). The major source of alginate is found in the cell walls and in the intracellular spaces of brown seaweed. The alginate molecules provide the plant with both flexibility and strength, which are necessary for plant growth in the sea. The first scientific studies on the extraction of alginates from brown seaweed were made by the British chemist E.C. Stanford who found that the extracted substance, which he named algin, possessed several interesting properties^29^, including the ability to thicken solutions, to make gels, and to form films. Sodium alginate is the main form of alginate. Other types of alginate include alginic acid, calcium, ammonium and potassium salts, and propylene glycol alginate, an ester of alginic acid.

**Figure 5:**
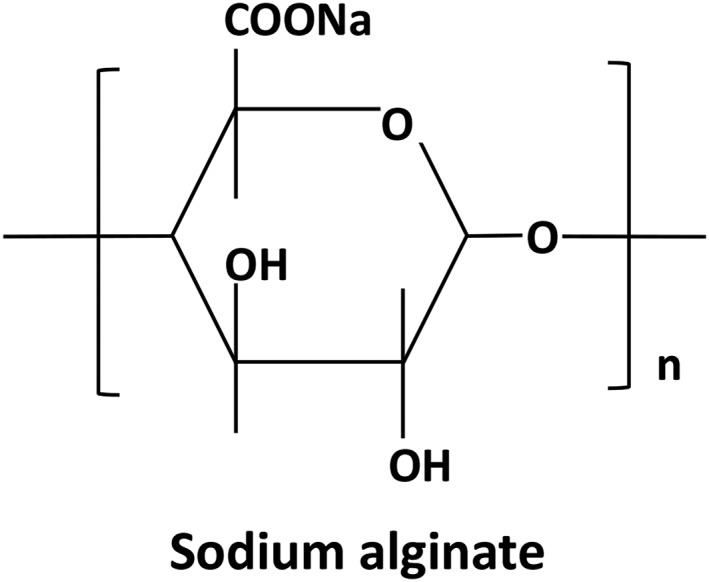
Chemical structure of sodium alginate, which is composed of consecutive G residues (GGGGGG), consecutive M residues (MMMMMM), and alternating M and G residues (GMGMGM).

Alginates are linear unbranched polymers containing β‐(1 → 4)‐linked D‐mannuronic acid (M) and α‐(1 → 4)‐linked L‐guluronic acid (G) residues. The blocks are composed of consecutive G residues (GGGGGG), consecutive M residues (MMMMMM), and alternating M and G residues (GMGMGM). Only the G‐blocks of alginate are believed to participate in intermolecular cross‐linking with divalent cations (e.g. Ca2+) to form hydrogels. The composition (i.e. M/G ratio), sequence, G‐block length, and molecular weight are critical factors affecting the physical properties of alginate and its resultant hydrogels^30^. The ability of alginates to form soft hydrogels with calcium ions forms the basis for a wide variety of applications. The structure of sodium alginate is shown in Fig. 5.

Sodium alginate slowly dissolves in cold water, forming a viscous, colloidal solution. It is insoluble in alcohol and hydroalcoholic solutions in which alcohol content is greater than 30% by weight. It is also insoluble in other organic solvents such as chloroform and ether and in acids where the pH of the resulting solution falls below 3.0.

Although the biocompatibility of alginates has been extensively evaluated *in vitro* as well as *in vivo*, there is still some debate regarding the impact of the alginate composition. Much of this confusion is likely related to various levels of purity in the alginates studied in various reports. One study suggests that as long as the alginate is purified, it is biocompatible^31^. However, the study of Gomez *et al*.^32^ indicates that the following two factors were more important for biocompatibility: M/G content and molar mass, probably *via* their influence on the physical properties (stiffness and swell ability) of the resulting gels.

#### *Carboxymethylcellulose*

**Figure 6:**
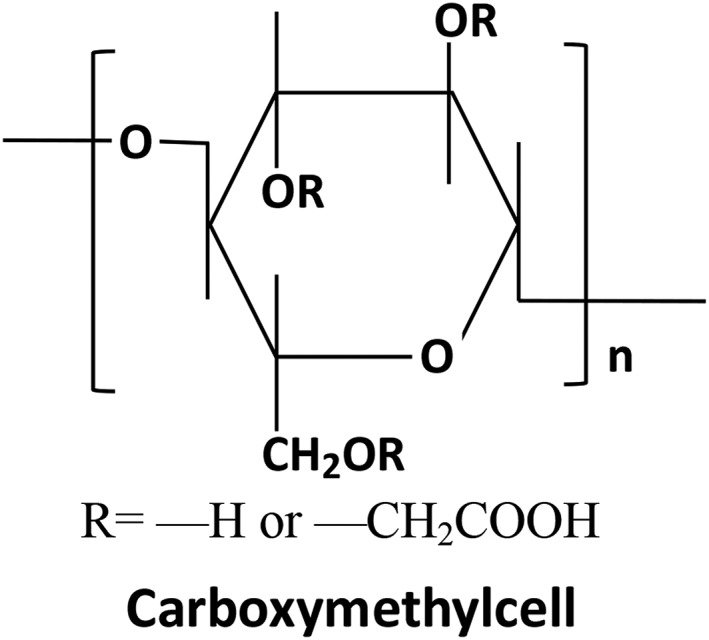
Chemical structure of carboxymethylcellulose (CMC) and its basic structure is a (1–4) d glucopyranosyl polymer.

Carboxymethylcellulose (CMC) is widely used in the pharmaceutical industry. It is a vegetable cellulose derivative obtained by the action of chloroacetic acid on cellulose in an alkaline medium, whereby hydroxyl functions are substituted by carboxymethyl groups. The basic structure is a (1–4) D glucopyranosyl polymer. The chemical structural of CMC is shown in Fig. 6.

Carboxymethylcellulose is a white to off‐white, odorless, and slightly hygroscopic powder with physiological inertia. It is soluble in water at all temperatures but practically insoluble in organic solvents. In addition, CMC is a stabilizing, emulsifying, thickening, binding, hydrophilic agent that can retain water and form a protective film. When dissolved or dispersed in water, it could increase its viscosity and contribute to form suspensions (from fluids to gels). Because CMC has good tissue compatibility and no toxic side effects on the body, it can be used as a biological material for implantation *in vivo* 33.

The role of CMC anti‐adhesion has been affirmed. The most important and widely recognized mechanism for its anti‐adhesion is the biomechanical isolation. CMC can also weaken the activity or proliferation of fibroblasts, prevent fibrin deposition on the damaged serosal surface^34^, prevent the removal of plasminogen from the wound surface, and increase its activation effectiveness^35^. Adanali *et al*.^36^ believe that the mechanism of CMC prevention of joint adhesion are exerting physical barriers, mitigating different inflammatory changes by exerting potential anti‐inflammatory effects after bone injury, including reducing joint capsule thickening and loss of adjacent tissue extensibility. The CMC prepared by Bae *et al*.^37^, which dissolves pure a‐cellulose in an alkaline solution, has a hemostatic effect, and it is believed that CMC can reduce adhesion by regulating the urokinase‐type plasminogen activator and its cellular receptor.

#### *Type I Collagen*

**Figure 7:**
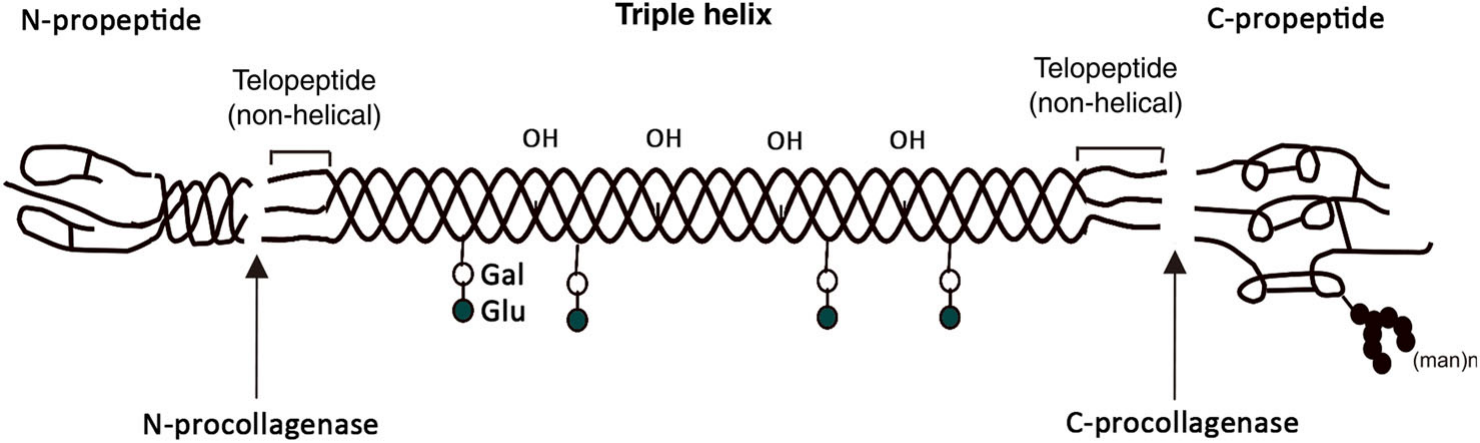
Structure of type I collagen, a right‐handed triple helix composed of two identical a1(I)‐chains and one a2(I)‐chain.

Collagen is a type of fibrous, macromolecular protein found in all mammals. It is secreted by connective tissue cells and other types of cells (such as liver, lung, spleen, and brain tissue cells) in mammals. As a major component of bones and skin, they are the most abundant protein in mammalian cells, accounting for approximately one‐quarter of total cellular proteins^38^. At present, 27 kinds of collagen have been found. The different collagen types are characterized by considerable complexity and diversity in their structure, splice variants, the presence of additional, non‐helical domains, assembly, and function. Despite the rather high structural diversity among the different collagen types, all members of the collagen family have one characteristic feature: a right‐handed triple helix composed of three a‐chains^39^. The type I collagen triple helix is usually formed as a heterotrimer by two identical a1(I)‐chains and one a2(I)‐chain^40^, whose structure is shown in Fig. 7.

Type I collagen is the most abundant collagen and is reported in many studies. It forms more than 90% of the organic mass of bone and is the major collagen of tendons, skin, ligaments, cornea, and many interstitial connective tissues, with the exception of very few tissues, such as hyaline cartilage, brain, and vitreous body. In most organs and notably in tendons and fascia, type I collagen provides tensile stiffness, and in bone, it defines considerable biomechanical properties concerning load bearing, tensile strength, and torsional stiffness in particular after calcification^41^.

Type I collagen contributes to the entrapment, local storage, and delivery of growth factors and cytokines, and plays an important role during organ development, wound healing, and tissue repair^42^. Furthermore, some additional features of collagens, such as biodegradability, low immunogenicity, inducing expression of BMP‐2 receptor^43^, and the possibilities for large‐scale isolation make them interesting compounds for medical application.

#### *Poloxamer 407*

Poloxamer copolymer is a tri‐block copolymer consisting of a central hydrophobic polyoxypropylene (PPO) block flanked by two hydrophilic polyoxyethylene (PEO) blocks (PEOx–PPOy–PEOx), whose chemical formula is HO[CH2‐CH2O]_*x*_[CH(CH3)‐CH2O]_*y*_[CH2‐CH2O]_*x*_OH, and *y* is higher than 14. It has a molecular weight of 1000 to more than 16 000, and is soluble in aromatic solvents and insoluble in ethylene glycol, kerosene, and mineral oil. Moreover, it is stable to acid, alkali, and metal ions^44^.

Poloxamer 407 is composed of approximately 70% ethylene oxide and 30% polypropylene oxide. Its aqueous solution with a concentration of 20%–30% has the property of reverse thermal gelation^45^. That is to say, it exists as a liquid at refrigerated temperature (4–5°C) but gels at physiological temperature. Bohorquez *et al*. indicate that when the critical micelle temperature is reached, the hydrophobic PPO block on the polymer chain is dehydrated and the poloxamer molecules aggregate in aqueous solution to form spherical micelles with dehydrated PPO chains as the core and hydrated expanded PEO chains as the outer shell^46^. With the increasing of temperature, gelation occurs due to the aggravation of entanglement and stacking between micelles. The temperature of solution–gel conversion is affected by the ratio of PEO/PPO, polymer concentration, and electrolytes in the solution. Some small molecules can change the phase transition temperature and gel strength of Poloxham 407 solution. The study by Yong demonstrated that diclofenac sodium significantly increased the gelation temperature and weakened the gel strength and bioadhesive force of Poloxamer 407, while sodium chloride did the opposite^47^.

Poloxamer 407 is a polymer material that cannot be excreted by the kidneys, so it is preferentially taken up by liver tissue, which may be one of the reasons for changing lipid metabolism. Poloxamer 407 interferes with the catalytic activity of 3‐hydroxy‐3methylglutaryl coenzyme A reductase required for cholesterol biosynthesis, while altering the release of heparin and intracellular lipoproteinase. Hyperlipidemia is caused by intraperitoneal administration of poloxamer 407 0.5–1 g·kg^−1^. Changes in lipid metabolism are still controversial and further research is required. Long‐term (1 year) administration of mouse poloxamer 407 did not reveal an effect on total cholesterol or alanine/aspartate activity. Poloxamer 407 did not cause an increase in morbidity or mortality compared to control mice.

### *Low Molecular Carriers Materials*

The low molecular carriers mainly include glycerol, calcium sulfate, and bioactive glass. They all have good plasticity, could be arbitrarily shaped mixed with DBM, and could help with osteogenesis due to their unique characteristics.

#### *Glycerol*

**Figure 8:**
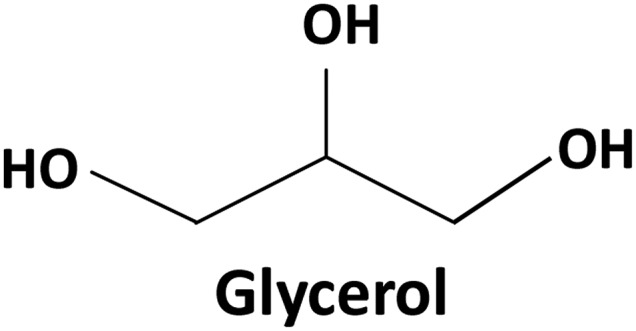
Chemical structure of glycerol.

Glycerol was initially discovered by Schell in 1779. Glycerol is a colorless, transparent, odorless, sweet organic compound with a chemical structure, which is shown in Figure 8. It is a clear and viscous liquid, mixed with water and alcohol, amines, and phenols in any proportion, and the aqueous solution is neutral. It has good biocompatibility and viscosity and is one of the commonly used viscous carriers for DBM.

#### *Calcium Sulfate*

Calcium sulfate (CS) is commonly referred to as gypsum and is a common mineral consisting of calcium sulfate dihydrate (CaSO_4_ • 2 H_2_O). CS and its products differ in purity and form, but a major feature is that water can be removed under controlled heat to form calcium sulfate hemihydrate, also known as plaster of Paris. This process is referred to as calcinatio^48^.:

(1) $${\text{CaSO}}_{4}\cdot 2H_{2}O\;\underset{\to }{{\text{CaSO}}_{4}}\cdot ½\;H_{2}O+1\;½\;H_{2}O.$$

Calcium sulfate hemihydrate has plasticity and *in‐situ* self‐curing properties. It can be made into various shapes according to the filling part, which is very suitable for filling and repairing bone defects. The setting of calcium sulfate hemihydrate is influenced by the milieu where it occurs. It is commonly reported that the addition of inorganic salts to CS, such as sodium chloride and potassium sulfate, could accelerate the setting reaction by increasing the density of the seed crystals^49^.

When the hemihydrate is mixed with water, Equation 1 is reversed in a mild exothermic reaction:

(2) $${\text{CaSO}}_{4}\cdot ½\;H_{2}O+1\;½\;H_{2}O\underset{\to }{{\text{CaSO}}_{4}}\cdot 2H_{2}O+\text{Heat}.$$

Because the hemihydrate is insoluble in water, a suspension is initially formed. As it slowly dissolves, a two‐phase suspension of hemihydrate particles in saturated aqueous solution exists. When the hemihydrate solution becomes supersaturated with dihydrate, dihydrate crystals nucleate in the suspension. Nucleation and crystal growth continue until the solution is no longer saturated, leading to further dissolution of the hemihydrate. Alternating dissolution and precipitation continues, with growth of existing crystals or nucleation of new crystals.

Calcium sulfate offers many advantages as it presents a structure similar to bone. It is osteoconductive^50^, inexpensive, and available in different forms (hard pellets and injectable fluids)^51^. The exact mechanisms through which calcium sulfate may enhance osteogenesis are unknown. It is possible that calcium ions are released during dissolution of calcium sulfate. Local increases in calcium ion concentration may affect osteoblast genesis and function, and they may act as the stimulus to osteoblast differentiation^52^. Other studies report that calcium sulfate has a crystalline structure that is osteoconductive, onto which bone capillaries and perivascular mesenchymal tissue can invade^53^. Walsh *et al*.^54^ report that locally altered pH may also play a role in osteogenesis around calcium sulfate implants. This increased acidity as the material dissolves can demineralize adjacent bone and release matrix‐bound bone growth factors that stimulate bone formation.

Biocompatibility is a *sine qua non* for implantable materials and is the result of complex interactions at the host–material interface^55^. The lack of significant host responses subsequent to implantation is an important characteristic of biocompatible materials. Many investigators have observed minimal inflammatory responses subsequent to implantation of CS^56^, ^57^, ^58^, ^59^.

#### *Bioactive Glass*

Bioactive glass is defined as a glass designed to elicit specific physiological responses^60^. In 1969, the first bioactive glass (Bioglass 45S5) was developed by L. Hench^61^, and it has been in clinical use since 1985. Many bioactive glasses consist of the same components, in slightly different concentrations (some representative bioactive glass compositions are shown in Table 1).

**Table 1:** Compositions of various bioactive glasses (wt%)

	SiO_2_	CaO	Na_2_O	K_2_O	MgO	P_2_O_5_	B_2_O_3_
45S5	45.0	24.5	24.5	—	—	6.0	—
13–93	53.0	20.0	6.0	12.0	5.0	4.0	—
13‐93B1	34.4	19.5	5.8	11.7	4.9	3.8	19.9
13‐93B3	‐	18.5	5.5	11.1	4.6	3.7	56.6
6P53B	52.7	18.0	10.3	2.8	10.2	6.0	—
58S	58.2	32.6	—	—	—	9.2	—
70S30C	71.4	28.6	—	—	—	—	—
P_50_C_35_N_15_	—	19.7	9.3	—	—	71.0	—

Bioactive glasses are extremely biocompatible. They do not evoke an inflammatory response when implanted into human or animal models. Injecting large doses of bioactive glass intramuscularly or subcutaneously had no adverse effect in a murine model^62^.

Bioactive glass is osteoconductive and osteoinductive and it can form a tight chemical bond with bone^63^. When exposed to real or simulated body fluids, dissolution of the bioactive glass surface is seen releasing Ca, P and Si ions because of its own special chemical composition. Dissolution and repolymerization of silica occur to form a silica gel on the surface. Amorphous calcium phosphate nucleates and grows in and on the SiO_2_‐rich layer. With time, the CaO‐P_2_O_5_ mineral incorporates carbonate and hydroxyl species from the ambient fluid, and hydroxycarbonate apatite (HCA) crystallizes. This layer is necessary for bone bonding^64^. This is similar to the process seen in hydroxyapatite.

It has been reported that bioactive glass can upregulate some essential genes for new bone formation such as insulin‐like growth factor (IGF‐II) and vascular endothelial growth factor (VEGF), in which IGF‐II could induce osteoblast proliferation, and VEGF could promote angiogenesis, which is required for new bone formation^65^.

## Commercially Available Demineralized Bone Matrix Products

There are many commercially available DBM products that have been used as a bone graft extender or as a bone graft substitute for a wide range of trauma‐related and orthopaedic‐related indications. The carriers and processing methods of different DBM products are different, which may have a certain impact on the osteogenic ability of the products. DBM products come in various forms, including sponges, strips, injectable putty, paste, and paste infused with chips^66^. These various forms also affect the products’ ability to serve as graft extenders, enhancers, or substitutes. Product names of common commercially available DBM products are shown in Table 2.

**Table 2:** Commercially available demineralized bone matrix (DBM)

Product	Source company	DBM(%)	Carrier	Form	Indication
Accell Connexus	Integra	70	Poloxamer reverse phase medium	Putty	Bone void filler/bone graft extender
Accell Evo3	Integra	70	Poloxamer reverse phase medium and cancellous bone chips	Putty	Bone void filler
Accell TBM	Integra	100	No carrier	Strip	Bone void filler/bone graft extender
AlloCraft	Stryker	80	Acellular matrix	Paste	Bone void filler
AlloFuse	Allosource	36 (putty), 29 (gel)	Reverse phase medium	Putty, gel	Bone void filler/bone graft extender
Allomatrix	Wright Medical	40 to 86	Calcium sulphate	Paste	Bone void filler/bone graft extender
AlphaGRAFT	Alphatech	80	Acellular matrix	Paste	Bone void filler
Altiva	Exactech	ND	Gelatin	Paste	Bone void filler
BioSet	Penta Biomedical	24	Porcine gelatin	Paste, strip, disc, with or without cancellous bone chips	Bone void filler
DBX	Medtronic	31 (putty), 26 (paste), 35 (mix), 45 (strip)	Hyaluronic acid	Putty, paste, mix, strip	Bone void filler
DynaGraft III	Integra	ND	Poloxamer reverse phase medium	Putty, gel	Bone void filler/bone graft extender
Grafton	Osteotech	17 to 31	Glycerol	Paste, strip	Bone void filler/bone graft extender/bone graft substitute
InterGro	Zimmer Biomet	40 (putty), 35 (paste)	Lecithin	Putty, paste	Bone void filler/bone graft extender
NanoFUSE	Amend Surgical	ND	45S5 bioactive glass	Putty	Bone void filler
Optefil	Exactech	24	Gelatin	Paste	Bone void filler
Opteform	Exactech	ND	cortical and cancellous bone chips suspended in collagen‐gelatin	Paste	Bone void filler
Optium	LifeNet Health	ND	Glycerol	Putty, gel	Bone void filler
OrthoBlast	Integra	ND	Poloxamer reverse phase medium	Paste	Bone void filler/bone graft extender
OrthoBlast II	Integra	ND	Poloxamer reverse phase medium	Putty, paste	Bone void filler/bone graft extender
Osteofil	Medtronic	24	Collagen	Paste, strip	Bone void filler
OsteoSelect	Bactarin International	74	Carboxymethylcellulose, phosphate buffered saline	Putty	Bone void filler
Progenix Plus	Medtronic	60	Type‐1 bovinecollagen and sodiumalginate	Putty	Bone void filler/bone graft extender/bone graft substitute
Progenix Putty	Medtronic	70	Type‐1 bovinecollagen and sodiumalginate	Putty	Bone void filler/bone graft extender/bone graft substitute
PRO‐STIM	Wright Medical	40	Calcium sulfate and calcium phosphate	Paste, putty	Bone void filler
VIAGRAF	Medtronic	ND	Glycerol	Paste, strip	Bone void filler

ND, no data available

### *Grafton*

Osteotech, in the United States, initially used glycerol as a carrier mixed with DBM to make Grafton, which has a DBM content of 17%–31%. It has been experimentally and clinically proven that Grafton has good biodegradability and arbitrary plasticity, and can be combined with growth factors or bone marrow stem cells.

Grafton was studied as a bone graft extender for posterolateral spinal fusion in a randomized controlled trial (RCT) by Cammisa *et al*. in 2004^67^. In 120 patients, posterolateral lumbar fusions were carried out with pedicle screw fixation and one side of the spine was grafted with autograft (17.2 standard deviation [SD] 9.7 mL), while the contralateral side was grafted with autograft and Grafton (17.2 SD 9.7 mL, mixed 1:2). Two years later, autograft with Grafton resulted in fusion in 42 cases (52%) and autograft alone resulted in fusion in 44 cases (54%). Kang *et al*.^68^ performed an RCT of 46 patients undergoing lumbar fusion surgery. Grafton was mixed with local bone or autogenous iliac bone at a ratio of 2:1 as bone graft material and followed up for 2 years. There was no significant difference in fusion rate and spinal function between the two groups. The authors concluded that Grafton is an effective bone graft extender and could be used in combination with local bone as a safe and effective spinal fusion method. In another prospective cohort study^69^, patients undergoing instrumented posterolateral lumbosacral spinal fusion were grafted with Grafton and aspiration of bone marrow (19 cases), Grafton and autologous bone (27 cases), or autologous bone alone (27 cases). All groups showed similar fusion rates after 2 years’ follow‐up (63%, 70%, and 67%, respectively). These studies provide evidence that Grafton can be used as a bone graft extender for lumbar spinal fusion.

Grafton was used in an RCT by An *et al*.^70^, which included 77 patients who underwent anterior cervical fusion. Grafton was combined with allografts and compared with autografts alone. Nonunion occurred in 46% of the patients who were grafted with Grafton and allografts, while in only 26% of patients who received an autograft (P = 0.11), suggesting that the combination of Grafton and allograft resulted in a higher rate of nonunion. Elsawaf *et al*.^71^ described completely filling the polyether ether ketone (PEEK) cage with Grafton in anterior cervical discectomy and fusion in a case series of 20 patients. The mean Cobb angle improved (3.4° pre‐operatively *vs*. 14.5° postoperatively) and Japanese Orthopaedic Association (JOA) myelopathy scores and neck disability index also subsequently improved after surgery. Park *et al*.^72^ used PEEK cages containing autologous bone chips and Grafton for cervical fusion in 31 patients. One year later, the overall fusion rate was 97%. Both the visual analogue scale (score for neck and arm pain) and the modified JOA scoring system for myelopathy were significantly improved. These studies indicate that the role of Grafton is uncertain as a bone graft extender for cervical spinal fusion.

Grafton was the only DBM product used for thoracic fusions. In a retrospective cohort, Park *et al*.^72^ used Grafton in patients who underwent anterior thoracic discectomies and compared their results with using morselized cancellous allografts. On the final radiographs, the allograft group fusion rate was 82% and the Grafton group fusion rate was 92%. There was no significant difference between the two groups. This study provides evidence that Grafton could be used as a bone graft substitute for thoracic spinal fusion.

Cheung *et al*.^73^ used Grafton as a bone graft extender, which was mixed with cancellous allografts to filled bone defects encountered in periarticular fractures of the tibia, fibula, femur, humerus, forearm, and acetabulum. Fracture healing occurred in 69% of the patients who received Grafton (n = 13). Grafton was also used to enhance cancellous allografts in two tibial stress fractures treated by drilling and bone grafting^74^, and for reconstructing large segmental bone defects of the tibia (n = 2^75^. and humerus (n = 1)^76^, using a titanium mesh cage filled with Grafton and cancellous allograft chips. These studies provide evidence that Grafton could be used in combination with allograft as a bone graft extender or enhancer to treat bone defects during fracture surgery.

Hierholzer *et al*.^77^ retrospectively analyzed 78 patients with nonunion of the tibia, of which 45 patients were treated with autogenous iliac bone transplantation and 33 patients were treated with Grafton. The healing rates of the two groups reached 100% and 97%, respectively. It is believed that Grafton can effectively promote bone healing, and the postoperative complications are lower than those in the ICBG group, which can be used as a treatment standard. In a case report^78^, Grafton was used to treat a non‐displaced coracoid fracture. After screw fixation, the nonunion site was debrided and successfully grafted with Grafton. These studies provide evidence that Grafton could be used as a bone graft substitute or bone graft extender to treat nonunion.

Furthermore, solitary bone cysts in children could be treated with Grafton. After filling the defects with Grafton in 7 cases, a continuous decrease in radiographic bone transparency was observed over a period of 2 years^79^. This study indicates that Grafton could be used as a bone graft substitute to treat solitary bone cysts.

Although Grafton has been widely used clinically, glycerol is water‐soluble, unstable, and potentially toxic in large‐scale use, and there is still some controversy about its use. To test the toxicity of Grafton, Wang *et al*.^80^ performed *in vivo* experiments on mice, and the results showed that the median lethal dose was 0.004 69 mL/g. It is recommended that the clinical application dose should not exceed 2 mL/kg. However, no serious adverse reactions related to glycerol have been found in clinical studies, adverse event reports, and published literature on Grafton. Therefore, from the previous clinical experience, glycerol toxicity should be considered unlikely^81^. In addition, Ziran *et al*.^82^ treated 25 patients who were smokers with fractures with Grafton, and found that the fracture healing rate was only 52% in the later follow‐up. Therefore, Grafton should be used carefully in the treatment of smokers with bone graft.

### *Allomatrix*

Allomatrix is manufactured by Wright Medical of the UK with calcium sulfate as a carrier. It has been widely used in clinical applications such as spinal fusion and trauma surgery because of its excellent osteoconductivity and degradability; it provides stable structural support for the growth of new bone.

Allomatrix has been used in posterolateral lumbar fusions. A case‐control study by Fu *et al*. showed that Allomatrix and autologous bone resulted in comparable fusion rates when used with hydroxyapatite/tricalcium phosphate granules: 81% and 86%, respectively^83^. Sapkas *et al*.^84^ described a retrospective case series following 32 patients who underwent posterior lumbar interbody fusion with Allomatrix, and clinical and radiological scores improved significantly with the mean follow‐up of 36 months (range, 18 to 42 months). At the latest follow‐up, the mean Oswestry Disability Index improved from 52% to 22%. The mean Roland–Morris Disability Questionnaire improved from 52% to 29%, while >90% of the operated levels were fused. In another retrospective case serie^85^. of 65 patients who underwent lumbar fusion by using Allomatrix mixed (1:1) with iliac crest bone, radiological follow‐up showed an improvement in the Lenke scores: 3.7 after 1 month to 1.6 after 12 months. These studies provide evidence that Allomatrix may be used as a bone graft extender for lumbar spinal fusion.

Allomatrix has also been used to treat distal radial fractures. Agostino *et al*.^86^ performed an RCT of 50 patients, in which unstable distal radial fractures were treated by operative fixation with Kirschner wires, with (*n* = 24) or without (*n* = 26) augmentation of the fracture site with Allomatrix. The physical and radiological outcomes did not show any significant difference in wrist function, speed to recovery, union rate, and complication rate after 1‐year follow‐up. In another RCT of 44 patients with femoral fractures who were treated with open reduction and internal fixation with (*n* = 33) or without (*n* = 11) Allomatrix, after 22 months of follow‐up, there was no significant difference in healing rate between the two groups. In the Allomatrix group, there were 5 cases of wound nonunion after surgery. Allomatrix has been used for primary treatment of fresh bone defects caused by small‐caliber gunshot wounds in the hand. In a retrospective case series of 12 patients, 11 bone defects healed without further intervention and 1 defect required a second bone grafting procedure^87^. These studies provide evidence that Allomatrix is not an ideal bone graft substitute to treat unstable fractures that have already been treated with internal fixation. It may even lead to complications such as wound nonunion.

Allomatrix, mixed with cancellous allograft chips, was used to treat 41 atrophic or avascular nonunions, which were located in the femur, radius, tibia, and humerus^88^. It was found that the secondary infection rate from drainage tubes was 51%; the deep infection rate was 34%, and the treatment failure rate was 34%. Therefore, the incidence of complications of Allomatrix was too high for it to be recommended for bone nonunion treatment, especially when there is a large volume defect or any previous infection of the focus. In addition, Allomatrix has been used to graft bone defects resulting after nonunion (n = 35)^89^. Allomatrix was mixed (1:3) with calcium sulphate pellets, and after 7 months, 85% of the grafted nonunions were healed. The abovementioned studies provide evidence that Allomatrix should not be used in the treatment of nonunion of bone, especially when there is a large volume defect or any previous infection of the focus.

Allomatrix has been used to treat benign bone tumors in the tibia (n = 17), humerus (n = 11), fibula (n = 3), and radius (n = 2)^89^. Allomatrix was mixed (1:3) with a calcium sulphate bone substitute(Osteoset) to fill defects. After 7 months, 93% of the bone defects were healed. Tumor recurrence was seen in 3 cases, and 1 wound infection required antibiotic treatment. In addition, in a study that investigated the treatment of 98 benign bone tumors located in the tibia, humerus, femur, and pelvis with various bone grafts, Allomatrix was used in 34 of the grafting procedures but no Allomatrix‐specific outcomes were reported^90^. These studies identify that Allomatrix could be used as a bone graft substitute with or without a calcium sulphate bone substitute to treat benign bone tumors.

### *Osteofil*

Osteofil is an injectable DBM product with porcine collagen as a carrier, in which DBM content is 24%. The common form of Osteofil is injectable paste and moldable strips, which can be arbitrarily changed in shape to fill bone defects. Due to its excellent osteoconductivity, osteoinductivity, and degradability, it has been widely used in the clinic.

Epstein *et al*.^91^ used a 1:1 mixture of Osteofil and autologous lamina for lumbar fusion, including 95 cases of primary fusion and 45 cases of secondary fusion. After 3 years of follow‐up, 93 (97.9%) of the patients with primary fusion had stable spinal fusion, and only 2 (2.1%) had unstable or pseudo articular joints on average 8 months after surgery. Of the patients with secondary fusion, 43 (95.6%) had stable spinal fusion, and 2 (4.4%) had instability on average 10 months after surgery. It could be concluded that Osteofil was an effective bone graft expander with autologous bone for lumbar spinal fusion.

Osteofil was mixed with autologous bone (n = 11) in a study by Epstein^92^, and this study also included 24 patients in which Vitoss (β‐tricalcium phosphate) was mixed with autologous bone. Radiological follow‐up showed that all levels were fused after an average of 5.2 months. Less than 50% of the original fusion mass remained visible on 2D‐CT scans after 6 months in 64% of fusions grafted with Osteofil, compared with 21% of fusions grafted with Vitoss, which suggested a quicker resorption rate of Vitoss. This study provides evidence that Osteofil may be used as a bone graft extender with autologous bone for cervical spinal fusion.

### *DBX*

DBX manufactured by Medtronic is a DBM product with HA as a viscous carrier. DBX can be used to promote osteogenesis and repair bone defects, but it can only be used for stable bone defects due to lack of structural strength for load‐bearing applications.

DBX has been used to treat sternal segment dislocations by Divisi *et al*.^93^ Eight patients with sternal segment dislocations were treated with titanium screws and DBX. Use of titanium crews and DBX reduced the length of hospitalization, and led to rapid functional recovery and excellent aesthetic results according to the authors. This study provides evidence that DBX could be used as a bone graft substitute to treat fractures of the sternum.

DBX has also been described in a case report, showing the successful treatment for subtrochanteric nonunion of an 11‐year‐old patient with an adult proximal humeral locking plate and additional grafting with DBX^94^. This study provides evidence that DBX could be used as a bone graft substitute to treat nonunion.

DBX was used as a graft in the treatment of enchondromas. Kwok *et al*.^95^ and Dietz *et al*.^96^ reported small case series of five and two patients, respectively. No recurrence or pathological fractures were reported. These studies provide evidence that DBX could be used as a bone graft substitute to treat hand enchondromas.

### *Accell Connexus*

Accell connexus is an injectable DBM product with Poloxamer as a carrier, in which DBM content is 70%. Provided in a syringe, the putty is moldable and resists irrigation. The putty may be implanted directly from the syringe. Accell connexus has been used as a bone graft extender in lumbar fusions.

Schizas *et al*.^97^ used a mixture of Accell Connexus and iliac crest bone for lumbar fusion in 33 patients. Compared with 26 patients who used iliac crest bone alone, there was no significant difference between the fusion rate and postoperative function. This study provides evidence that Accell connexus could be used as a bone graft extender of iliac crest bone for lumbar spinal fusion.

## Summary and Outlook

After many years of research, DBM products have been increasingly used in clinical applications, and some therapeutic effects have been achieved. However, there are still several problems that need to be studied and discussed: (i) there is currently no DBM product that can combine the various conditions of ideal bone graft materials; and (ii) different materials, different methods, different reagents, and even the same conditions of batch processing of DBM products have differences in osteogenic activity. At present, there is no determination of the conditions under which the products have the best osteogenic capacity. Therefore, research efforts with DBM must be continued to expand clinical applications, produce validated utility, and demonstrate new options and opportunities to enhance clinical outcomes in bone repair. In the future, the combined use of DBM with other bone‐promoting substances such as autologous stem cells from bone marrow aspirates, seed cells, and growth factors may be an important research direction.

## Disclosure

This research did not receive any specific grant from funding agencies in the public, commercial, or not‐for‐profit sectors.
